# Nutrient exchange in arbuscular mycorrhizal symbiosis from a thermodynamic point of view

**DOI:** 10.1111/nph.15646

**Published:** 2019-01-21

**Authors:** Ingo Dreyer, Olivia Spitz, Kerstin Kanonenberg, Karolin Montag, Maria R. Handrich, Sabahuddin Ahmad, Stephan Schott‐Verdugo, Carlos Navarro‐Retamal, María E. Rubio‐Meléndez, Judith L. Gomez‐Porras, Janin Riedelsberger, Marco A. Molina‐Montenegro, Antonella Succurro, Alga Zuccaro, Sven B. Gould, Petra Bauer, Lutz Schmitt, Holger Gohlke

**Affiliations:** ^1^ SFB 1208 – Identity and Dynamics of Membrane Systems – from Molecules to Cellular Functions Heinrich Heine Universität Düsseldorf Universitätsstraße 1 40225 Düsseldorf Germany; ^2^ Centro de Bioinformática y Simulación Molecular (CBSM) Facultad de Ingeniería Universidad de Talca 2 Norte 685 Talca 3460000 Chile; ^3^ Institute for Pharmaceutical and Medicinal Chemistry Heinrich‐Heine‐Universität Düsseldorf Universitätsstraße 1 40225 Düsseldorf Germany; ^4^ Institute of Biochemistry Heinrich‐Heine‐University Düsseldorf Universitätsstraße 1 40225 Düsseldorf Germany; ^5^ Institute of Botany Heinrich‐Heine University Universitätsstraße 1 40225 Düsseldorf Germany; ^6^ Institute for Molecular Evolution Heinrich Heine University Universitätsstraße 1 40225 Düsseldorf Germany; ^7^ Instalación en la Academia Núcleo Científico Multidisciplinario Dirección de Investigación Vicerrectoría Académica Universidad de Talca 2 Norte 685 Talca 3460000 Chile; ^8^ Instituto de Ciencias Biológicas Universidad de Talca Avenida Lircay s/n Talca 3460000 Chile; ^9^ Centro de Estudios Avanzados en Zonas Áridas (CEAZA) Universidad Católica del Norte Avda. Larrondo 1281 Coquimbo Chile; ^10^ Life and Medical Sciences (LIMES) Institute University of Bonn Carl‐Troll‐Str. 31 53115 Bonn Germany; ^11^ Botanical Institute Cluster of Excellence on Plant Sciences (CEPLAS) University of Cologne 50674 Koln Germany; ^12^ John von Neumann Institute for Computing (NIC) Jülich Supercomputing Centre (JSC) & Institute for Complex Systems – Structural Biochemistry (ICS‐6) Forschungszentrum Jülich GmbH 52425 Jülich Germany

**Keywords:** computational cell biology, modelling, nutrient transport, plant biophysics, plant–fungus interaction

## Abstract

- To obtain insights into the dynamics of nutrient exchange in arbuscular mycorrhizal (AM) symbiosis, we modelled mathematically the two‐membrane system at the plant–fungus interface and simulated its dynamics.
- In computational cell biology experiments, the full range of nutrient transport pathways was tested for their ability to exchange phosphorus (P)/carbon (C)/nitrogen (N) sources.
- As a result, we obtained a thermodynamically justified, independent and comprehensive model of the dynamics of the nutrient exchange at the plant–fungus contact zone. The predicted optimal transporter network coincides with the transporter set independently confirmed in wet‐laboratory experiments previously, indicating that all essential transporter types have been discovered.
- The thermodynamic analyses suggest that phosphate is released from the fungus via proton‐coupled phosphate transporters rather than anion channels. Optimal transport pathways, such as cation channels or proton‐coupled symporters, shuttle nutrients together with a positive charge across the membranes. Only in exceptional cases does electroneutral transport via diffusion facilitators appear to be plausible. The thermodynamic models presented here can be generalized and adapted to other forms of mycorrhiza and open the door for future studies combining wet‐laboratory experiments with computational simulations to obtain a deeper understanding of the investigated phenomena.

## Introduction

In the soil, plants share their habitat with microorganisms (Jacoby *et al*., 2017) and both compete for the same nutrient resources. Nevertheless, microbial activity and plant productivity often strongly depend on each other, especially in nutrient‐poor ecosystems (Hodge *et al*., 2000). Quite frequently, plants engage in symbiotic interactions with microorganisms to improve nutrient uptake. More than 80% of land plants form associations with arbuscular mycorrhizal (AM) fungi, in which they greatly benefit from nutrients provided by the fungi, in particular phosphate and nitrogen (N). In return, plants provide the fungi with organic carbon (C) in the form of carbohydrates and fatty acids (Finlay, 2008; Smith & Read, 2008; Bravo *et al*., 2017; Jiang *et al*., 2017; Keymer *et al*., 2017; Kim *et al*., 2017; Luginbuehl *et al*., 2017). Despite substantial progress over recent years in the understanding of this bidirectional nutrient exchange (reviewed, for instance, in Lanfranco *et al*., 2016; MacLean *et al*., 2017; Roth & Paszkowski, 2017; Wang *et al*., 2017; Chen *et al*., 2018), several of its aspects still remain poorly understood.

Considering that each exchanged nutrient molecule needs to cross both the plant and the fungal plasma membrane, it might be hypothesized that the nutrient trade strongly depends on the dynamics of the two‐membrane system of the plant–fungus contact zone. Apart from concentration gradients, such a dynamic is governed by the electrical force, the strongest known force outside the subatomic world. Indeed, several nutrients are charged *per se* (e.g. H_2_PO_4_^−^, NO_3_^−^, NH_4_^+^, K^+^) or, if they are electrically neutral (e.g. sugars), they are often transported together with protons (H^+^).

**Figure 1:**
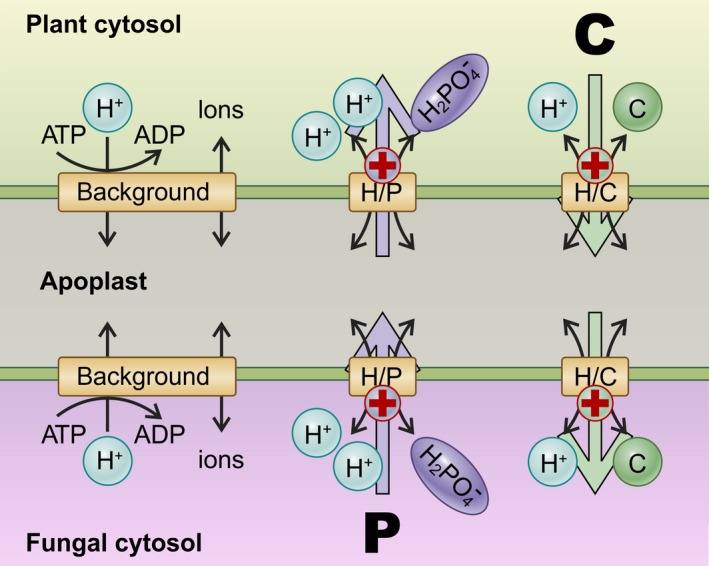
A minimal transporter network for phosphate/sugar nutrient exchange in arbuscular mycorrhizal (AM) symbiosis modified from Schott *et al*. (2016). In the minimal model, both plant and fungal plasma membranes contain proton‐coupled phosphate transporters (H/P), proton‐coupled sugar transporters (H/C) and H^+^‐ATPase‐driven background conductances. The small black arrows indicate possible transport directions of the transporters depending on the prevailing electrochemical gradients. The large coloured arrows indicate fluxes in AM symbiosis. The ‘+’ indicates that transport of the nutrient is accompanied by the transport of a positive charge across the membrane.

One previous study has approached the nutrient exchange in AM symbiosis from the perspective of membrane biophysics (Schott *et al*., 2016). A minimal transporter network was simulated in computational cell biology experiments, considering only those transporters that were experimentally shown at that time to be expressed during arbuscule formation: proton‐coupled phosphate and sugar transporters (H/P and H/C symporters) and an H^+^‐ATPase‐driven background conductance in both the plant and fungal membranes (Fig. 1). Despite its simplicity, this minimal model could explain many characteristics of the nutrient trade between the plant and fungus. For instance, it could explain – mechanistically – the microscopic exchange (i.e. transfer across the two membranes) of phosphate and sugar sources, which, in turn, also explained the macroscopic observation (i.e. at the organism level) of reciprocal rewards between the plant and fungus, when providing more sugar and more phosphate, respectively. In combination with basic microeconomic principles, it could furthermore explain the macroscopic observation that fertilization with mineral phosphate is detrimental for the stability of AM symbiosis. In this case, the plant roots acquire more phosphate directly from the soil and the phosphate provided by the fungus is of lesser value. Under these circumstances, the transporter network at the plant–fungus interface adjusts its parameters in a manner that cuts the fungus off from C provision.

Regardless of its predictive power, it is, however, not unequivocally clear whether the minimal model truly reflects the real situation or whether the accordance between computational simulations and experiments is just a coincidence and alternative scenarios are employed in AM symbiosis. It is still uncertain whether our knowledge on the transporter types involved in the nutrient exchange is complete. Phosphorus (P) and C sources, for instance, might be transported, not through proton‐coupled symporters alone, but also by diffusion facilitators; P could be transported via phosphate‐permeable anion channels (Dreyer *et al*., 2012) and C could be transported in neutral form as a sugar through sugar channels of the SWEET type (Sugar Will Eventually be Exported Transporter; Chen *et al*., 2010) or as organic acids through channels of the ALMT type (ALuminium activated Malate Transporter; Sharma *et al*., 2016). In this study, we tackled this issue by evaluating, in an unbiased manner, the thermodynamic features of general transporter network models for their use in nutrient exchange in AM symbiosis. While the model of Schott *et al*. (2016) was limited to prior knowledge on the transporters involved in AM symbiosis, we here consider all possible transporter types, even though they are only hypothetical, and assess whether they are suitable for the nutrient trade. The computational cell biology experiments covered all different possibilities of alternative and/or enlarged models for C, P and N exchange in ‘*what‐if*’ scenarios, and allowed an evaluation of their performance using the minimal model (Fig. 1) as a benchmark. As a result, we obtained a comprehensive view on the dynamics of nutrient exchange in AM symbiosis that rests on the grounds of first principle thermodynamics.

Notably, our predictions for an optimal transporter network coincide with the transporter set confirmed independently in wet‐laboratory experiments by others, indicating that the already discovered transporters are sufficient for optimal performance and suggesting that our knowledge of the fundamental transporter types involved in nutrient exchange in AM symbiosis is rather complete.

## Description

The modelling approach was essentially performed as described previously (Schott *et al*., 2016). The assessment of several fundamental features of the models is presented in Supporting Information Methods S1; for an explanation of any technical detail that is not repeated here, the reader is encouraged to read the article by Schott *et al*. (2016).

Following the mathematical description of all transporters, an *in silico* cellular system was programmed and computational cell biology (dry‐laboratory) experiments were performed using the vcell modeling and analysis platform developed by the National Resource for Cell Analysis and Modeling, University of Connecticut Health Center (Loew & Schaff, 2001). In all scenarios investigated in this study, the different fluxes rapidly reached system‐dependent steady‐state levels, which were independent of the starting conditions. These steady‐state fluxes between plant and fungus reflected the nutrient exchange between the two organisms.

### Mathematical description of transporter activities

#### Background current

The voltage dependence of the H^+^‐ATPase‐dominated background current was approximated by the sigmoidal function:

(Eqn 1) $$I_{\mathrm{background}}=I_{\mathrm{BG}}=I_{\mathrm{BGmax}}\cdot \frac{1-e^{-\left(V_{m}-V_{o}\right)\cdot \frac{F}{\mathrm{RT}}}}{1+e^{-\left(V_{m}-V_{o}\right)\cdot \frac{F}{\mathrm{RT}}}}.$$

Here, *RT*/*F* ≈ 25 mV is a factor composed of the gas constant, temperature and Faraday's constant, *V*_m_ is the voltage at the respective plasma membrane, *I*_BGmax_ is the maximal background current and *V*_0_ is the equilibrium voltage at which *I*_BG_ = 0. *V*_0_ can be influenced by the activity of the H^+^‐ATPase. A larger activity increases the efflux of positive charges and drives *V*_0_ to more negative voltages. Otherwise, *V*_0_ can also be influenced by the activation of ion channels. If, for instance, a K^+^ channel is activated, *V*_0_ is driven towards *E*_K_, the Nernst‐equilibrium voltage for K^+^. The effects of changes in *V*_0_ have been investigated previously (Schott *et al*., 2016).

#### Proton‐coupled symporters

The current–voltage characteristics of proton‐coupled H^+^/*X* cotransporters *I*_*H/X*_ reverse their direction at *E*_*H/X*_, *I*_*H/X*_(*E*_*H/X*_) = 0. *E*_*H/X*_ depends on the concentrations of protons and the cotransported molecule *X* at both sides of the membrane:

(Eqn 2) $$E_{H/X}=\frac{\mathrm{RT}}{F}\cdot \frac{n_{X}\cdot \log_{e}\frac{{\left[H\right]}_{\mathrm{apo}}}{{\left[H\right]}_{\mathrm{cyt}}}+\log_{e}\frac{{\left[X\right]}_{\mathrm{apo}}}{{\left[X\right]}_{\mathrm{cyt}}}}{n_{X}+z_{X}}.$$

[*H*]_apo_, [*H*]_cyt_, [*X*]_apo_ and [*X*]_cyt_ are the concentrations of protons and the substrate *X* in the apoplast and in the cytosol, respectively. *z*_*x*_ is the valence of the ion/metabolite *X*,* z*_*x*_ = 0 in the case of sugar or other neutral substrates, *z*_*x*_ = −1 in the case of dihydrogen phosphate (H_2_PO_4_^−^) and nitrate (NO_3_^−^); *n*_*x*_ is the number of protons transported per particle *X*. Without affecting the results qualitatively, the current–voltage dependence of H^+^/*X* cotransporters can be approximated by a linear function (Dreyer, 2017) according to the first‐order Taylor approximation:

(Eqn 3) $$I_{H/X}=G_{H/X}\cdot \left(V_{m}-E_{H/X}\right).$$

*G*_*H/X*_ is the membrane conductance for H^+^/*X* symport and is proportional to the expression level and the activity of the transporter. For *E*_*H/X*_, we set *n*_*x*_ = 1 for the transport of uncharged substrates and *n*_*x*_ = 2 for dihydrogen phosphate and nitrate transporters.

#### SWEETs

Sugar flux through SWEET channels was modelled as a diffusion process:

(Eqn 4) $$J_{\mathrm{SWEET}}={\text{Diff}}_{\mathrm{SWEET}}\cdot \left({\left[C\right]}_{{\mathrm{cyt}}^{\mathrm{plant}}}-{\left[C\right]}_{\mathrm{apo}}\right).$$

Diff_SWEET_ is the SWEET‐mediated permeability of the membrane and is proportional to the expression level and the activity of SWEETs. [*C*]_cyt_^plant^ and [*C*]_apo_ denote the sugar concentration in the plant cytosol and the apoplast, respectively.

#### NH_4_^+^ permeases

Ammonium transport through NH_4_^+^ channels was approximated by a linear function (first‐order Taylor approximation):

(Eqn 5) $$I_{{\mathrm{NH}}_{4}^{+}}=G_{{\mathrm{NH}}_{4}^{+}}\cdot \left(V_{m}-E_{{\mathrm{NH}}_{4}^{+}}\right),$$

with the membrane conductance for NH_4_^+^, $G_{{\mathrm{NH}}_{4}^{+}}$, which is proportional to the expression level and activity of the permease, and the Nernst equilibrium voltage for ammonium, $E_{{\mathrm{NH}}_{4}^{+}}$, which depends on the cytosolic and apoplastic ammonium concentrations [NH_4_^+^]_cyt_ and [NH_4_^+^]_apo_, respectively:

(Eqn 6) $$E_{{\mathrm{NH}}_{4}^{+}}=\frac{\mathrm{RT}}{F}\cdot \log_{e}\frac{{\left[{\text{NH}}_{4}^{+}\right]}_{\mathrm{apo}}}{{\left[{\text{NH}}_{4}^{+}\right]}_{\mathrm{cyt}}}.$$

#### NH_3_ permeases

Ammonia transport through NH_3_ channels was modelled as a diffusion process, which depends on the concentration difference of [NH_3_] between both sides of the membrane. Because, even at pH 7, > 99% of ammonia/ammonium exists in the form of NH_4_^+^:

(Eqn 7) $$\left[{\text{NH}}_{3}\right]=10^{\left(\mathrm{pH}-9.25\right)}\cdot \left[{\text{NH}}_{4}^{+}\right],$$

it is useful to express the [NH_3_] difference as a pH‐dependent [NH_4_^+^] difference:

(Eqn 8) $$J_{{\mathrm{NH}}_{3}}={\text{Diff}}_{{\mathrm{NH}}_{3}}\cdot \left({\left[{\text{NH}}_{3}\right]}_{\mathrm{cyt}}-{\left[{\text{NH}}_{3}\right]}_{\mathrm{apo}}\right)=D_{{\mathrm{NH}}_{3}}\cdot \left(10^{{\mathrm{pH}}_{\mathrm{cyt}}}\cdot {\left[{\text{NH}}_{4}^{+}\right]}_{\mathrm{cyt}}-10^{{\mathrm{pH}}_{\mathrm{apo}}}\cdot {\left[{\text{NH}}_{4}^{+}\right]}_{\mathrm{apo}}\right),$$

with the cytosolic and apoplastic ammonium and ammonia concentrations [NH_4_^+^]_cyt_, [NH_4_^+^]_apo_, [NH_3_]_cyt_ and [NH_3_]_apo_, respectively. The membrane permeability $D_{{\mathrm{NH}}_{3}}=10^{-9.25}\cdot {\text{Diff}}_{{\mathrm{NH}}_{3}}$ is proportional to the expression level and activity of the NH_3_ channel.

#### Anion channels

Anion transport through anion channels was approximated by a linear function (first‐order Taylor approximation):

(Eqn 9) $$I_{A^{-}}=G_{A^{-}}\cdot \left(V_{m}-E_{A^{-}}\right),$$

with the membrane conductance for *A* ^−^, $G_{A^{-}}$, which is proportional to the expression level and activity of the channel, and the Nernst equilibrium voltage for the anion, $E_{A^{-}}$, which depends on the valence, *z*_*A‐*_, and the cytosolic and apoplastic concentrations of the anion *A* ^−^, [*A* ^−^]_cyt_ and [*A* ^–^]_apo_, respectively:

(Eqn 10) $$E_{A^{-}}=\frac{\mathrm{RT}}{z_{A^{-}}\cdot F}\cdot \log_{e}\frac{{\left[A^{-}\right]}_{\mathrm{apo}}}{{\left[A^{-}\right]}_{\mathrm{cyt}}}.$$

It should be noted that the parameters *G*_*H/X*_, Diff_SWEET_, $G_{{\mathrm{NH}}_{4}^{+}}$, $G_{{\mathrm{NH}}_{3}}$ and $G_{A^{-}}$ are in fact composite parameters, which are not only proportional to the expression level of the respective transporter, but usually dependent in a complex manner on a variety of environmental parameters, including the membrane voltage, concentration of the transported substrate (*K*_m_ value) or kinase‐ and phosphatase‐dependent regulatory cascades. To cover all this complexity, we focused in our modelling approach on the fluxes in equilibrium and screened the composite parameters *G*_*H/X*_, Diff_SWEET_, $G_{{\mathrm{NH}}_{4}^{+}}$, $D_{{\mathrm{NH}}_{3}}$ and $G_{A^{-}}$ in the entire reasonable parameter interval (0|∞). With this precaution, any possible regulation of these composite parameters was implicitly considered (for further explanations, see also Methods S1). To dissect the dependence of a composite parameter on an environmental parameter, the obtained results need to be interpreted appropriately, for example, as for the sugar transfer from the plant *Zea mays* to the parasitic fungus *Ustilago maydis* (Wittek *et al*., 2017).

## Results

In this study, we asked which transporter types might perform best in the nutrient exchange in AM symbiosis. To avoid missing a transporter type, we approached this question in a general, unbiased manner and used a process of elimination. Considering that a nutrient can either be taken up or released and that, based on the electrogenic nature of membrane transport, each transmembrane transport process can be classified into three categories ((1) transport of a nutrient molecule together with a positive charge; (2) transport without transmembrane net charge movement; and (3) transport together with a negative charge), we modelled six (2 × 3) hypothetical ‘*what‐if*’ cases.

### Cases 1–3: nutrient uptake

In AM symbiosis, both plant and fungus employ H^+^‐ATPases to energize uptake processes. Therefore, the prevailing membrane voltage at their plasma membranes is negative (i.e. the electric potential on the inside is negative with respect to the outside). A transport process without transmembrane net charge movement could not benefit from this electrical energy. The uptake of nutrients together with a negative charge, such as the direct influx of phosphate, would be against this electrical gradient. By contrast, the transmembrane electrical gradient established by the H^+^‐ATPase strongly favours nutrient uptake together with a positive charge (as illustrated in the minimal network in Fig. 1; Schott *et al*., 2016). Therefore, it is not surprising that plants and fungi employ either cation channels (e.g. NH_4_^+^ permeases or K^+^ channels) or proton‐coupled symporters (with stoichiometries that guarantee a positive net charge transport) for nutrient acquisition.

### Case 4: nutrient release together with a negative charge

**Figure 2:**
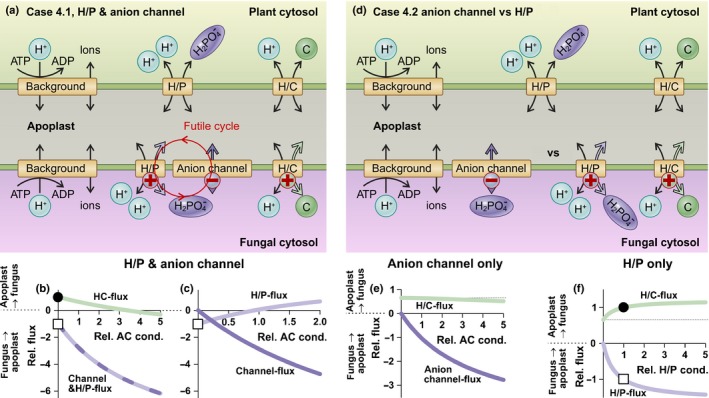
Hypothetical scenarios for the efflux of negatively charged nutrients via anion channels. (a–c) Additional phosphate efflux from the fungus via phosphate‐permeable anion channels. (a) The minimal model was enlarged by a phosphate‐permeable anion channel. (b) Dependence of the carbon (C) (green, H/C flux) and phosphorus (P) (dark and bright purple line, channel & H/P flux) fluxes across the fungal membrane on the conductance of the anion channel (rel. AC cond.). A positive value denotes an influx from the apoplast to the fungus, whereas a negative value reflects an efflux. The phosphate flux indicates the sum of the fluxes through the H/P symporter and anion channel. (c) Separate display of the phosphate flux through the H/P transporter (bright purple, H/P flux) and the anion channel (dark purple, channel flux). Please note that the coexistence of an anion channel and an H/P transporter establishes a futile cycle of phosphate at the fungal membrane. (d–f) Comparison of models containing only anion channels (e) or H/P transporters (f). (e) Dependence of the C (green, H/C flux) and P (dark purple, anion channel flux) fluxes across the fungal membrane on the conductance of the anion channel. (f) Dependence of the C (green, H/C flux) and P (bright purple, H/P flux) fluxes across the fungal membrane on the conductance of the H/P transporter. Note that, in (e), the H/C flux decays with increasing phosphate transporter conductance, whereas, in (f), it increases. The dotted lines indicate the H/C flux values in the absence of any phosphate transporter activity. The black dots and white squares in (b), (c) and (f) indicate the standard conditions of the reference model (Fig. 1). The ‘+’ (in a and d) indicates that transport of the nutrient is accompanied by the transport of a positive charge and ‘−’ indicates that it is accompanied by the transport of a negative charge across the membrane.

Here, we considered the hypothetical scenario that the fungus exports phosphate ions not only via a proton‐coupled phosphate transporter, but also via a phosphate‐permeable anion channel (Fig. 2a). We started our computational simulation with inactive anion channels (relative anion channel conductance = 0) and repeated the simulations with increased conductance levels. Without any anion channel activity, there was a constant flux of phosphate via the H/P symporters from the fungus to the plant (Fig. 2b, white square), accompanied by a constant flow of the C source (e.g. sugar) in the opposite direction (Fig. 2b, black dot).

With increasing channel conductance, the net flux of phosphate from the fungus to the apoplast and from the apoplast to the plant increased (Fig. 2b, dashed dark and bright purple line, channel & H/P flux), indicating that a phosphate‐permeable anion channel was indeed well suited for the release of phosphate from the fungus. Of course, the plant benefited from this increased P flux; the fungus, however, did not share this benefit. By contrast, the C flux from the plant to the fungus via the apoplast decreased with increasing channel activity (Fig. 2b, green, H/C flux). At a certain conductance level, the C flux was zero and even negative at higher activities, which means that the C flux direction was inverted; the fungus thus exported both P and C. Moreover, the coexistence of anion channels with proton‐coupled H/P transporters generated a futile cycling of phosphate (Britto & Kronzucker, 2006). Although the H/P transporter initially functioned as a phosphate exporter, it converted into an importer with increasing channel conductance (Fig. 2c, bright purple, H/P flux). The increasing phosphate efflux via the anion channel increased the apoplastic phosphate concentration, which, in turn, affected the operation mode of the proton‐coupled phosphate transporter. Above a certain phosphate concentration – the exact value was dependent on the membrane voltage, the pH gradient and the cytosolic phosphate concentration – the inward‐directed electrochemical proton gradient overcompensated the outward‐directed phosphate gradient and drove phosphate uptake. Thus, the fungus had to constantly invest additional energy via the H^+^‐ATPase to fuel the re‐uptake of phosphate. Therefore, the coexistence of H/P transporters and phosphate‐permeable anion channels in the fungal plasma membrane did not benefit the fungus.

Consequently, we explored a second scenario in which the anion channel was not added to the proton‐coupled H/P transporter, but replaced it, and compared this network with the benchmark model with the H/P symporter (Fig. 2d). Also, in this scenario, the anion channel worked well for phosphate release. The side‐effects, however, were similar to those reported for the first scenario. The fungus did not benefit from an increased phosphate release, but instead received less C from the plant (Fig. 2e, green, H/C flux, Fig. S1d–f). Thus, there was no reward for the fungus, but rather a penalty, if it provided phosphate via an anion channel (further details are provided in Fig. S1). By contrast, if the fungus provided phosphate via a proton‐coupled H/P symporter, it benefited from a higher C flux from the plant when the phosphate release was increased (Fig. 2f).

The fundamental reason why an anion channel was not well suited in a nutrient trade was the charge of the transported substrate. For each phosphate molecule that was released via the anion channel, a negative charge was transported across the membrane. The efflux of a negative charge depolarized the membrane and dissipated the electric gradient established by the H^+^‐ATPase, whereas the efflux of a positive charge – as in the reference network (Figs 1, 2f, S1a–c) – further strengthened this gradient (Dreyer *et al*., 2017). In steady‐state conditions, such as those considered here, the efflux of phosphate via H/P transporters across the fungal membrane was accompanied by the export of a positive charge. This supported the proton pump and provided an additional energy source for the inverse H/C flux. If, by contrast, phosphate was released via anion channels, this support was absent. Instead, another positive charge needed to be released (e.g. by pumping an additional proton out of the cell or by releasing K^+^) to maintain charge neutrality.

The results obtained for a hypothetical phosphate‐permeable anion channel at the fungal membrane can be generalized. It can be translated one‐to‐one to all scenarios, in which the fungus or the plant releases a nutrient together with a negative charge. One example might be the hypothetical release of C from the plant in the form of negatively charged organic acids via anion channels of the ALMT type. In this case, one can similarly conclude that it is not for the benefit of the plant to express such types of channel in the membrane facing the peri‐arbuscular space. Because of the negative charge net transport from the cytosol to the apoplast, the plant would compromise the desired uptake of phosphate (for further discussion, see also Notes S1).

### Case 5: nutrient release without transmembrane net charge movement

**Figure 3:**
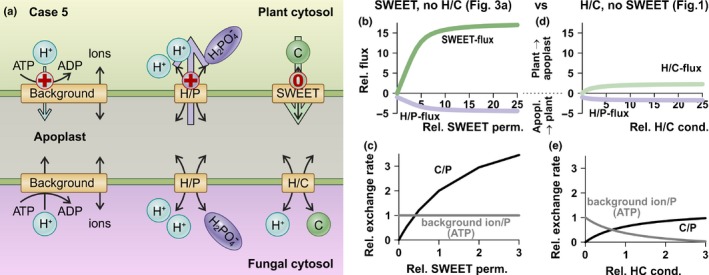
Hypothetical scenario for the electroneutral efflux of nutrients. (a) Compared with the minimal model (Fig. 1), the proton‐coupled H/C transporter was replaced by a SWEET diffusion facilitator. (b, c) Only SWEET, no plant H/C activity. (d, e) Only H/C, no SWEET activity. (b, d) Dependence of the carbon (C, green) and phosphorus (P, purple) fluxes on the permeability/conductance of the respective C transporter. (c, e) Dependence of the relative C/P (black) and background ion (ATP)/P (grey) exchange rates of the plant on the permeability/conductance of the respective C transporter. The large coloured arrows in (a) indicate the prevalent fluxes across the plant plasma membrane in arbuscular mycorrhizal (AM) symbiosis. The ‘+’ indicates that transport of the nutrient is accompanied by the transport of a positive charge across the membrane and ‘0’ that transport of the nutrient is electroneutral.

In this case, we tested the efflux of a C source from the plant by diffusion facilitators. We considered here sugar channels of the SWEET type (Chen *et al*., 2010) as an example (Figs 3, S2). The case of Fig. 3 would also cover scenarios in which, for instance, fatty acids are released by other electroneutral transport processes (Zhang *et al*., 2010; Gutjahr *et al*., 2012; Bravo *et al*., 2016; Li *et al*., 2016; Jiang *et al*., 2017; Keymer *et al*., 2017; Luginbuehl *et al*., 2017).

First, we extended the reference model (Fig. 1) with SWEETs (Fig. S2). This established a futile cycle of C efflux via SWEET and H^+^‐ATPase‐driven C influx via the H/C transporter (Fig. S2). The futile cycle, however, was not as severe as that shown in Fig. 2(a–c), because it affected mainly the energy status of the plant. There was no reduction in P uptake; instead, the P uptake was uncoupled from the C supply in a manner in which an increase in provided C did not result in increased P acquisition. Thus, the presence of both SWEETs and H/C transporters was not optimal in terms of energy and C use efficiency (Fig. S2).

We eliminated the futile cycle and replaced the plant H/C symporter by SWEET (Fig. 3). The performance of this model was compared with the reference network (Fig. 1). Interestingly, both scenarios were suited for a P/C trade between plant and fungus. With increasing plant sugar transporter permeability (SWEET) or conductance (H/C), the flux of sugars from the plant to the fungus, as well as the flux of phosphate in the opposite direction, increased (Fig. 3b,d). It further turned out that up to eightfold more sugar could be shuttled from the plant to the fungus with SWEET than with the H/C transporter. However, in return, the plant received just up to 2.6‐fold more phosphate. Thus, with increasing SWEET permeability, the plant had to provide more C for one P molecule, whereas the background ion flux per P remained stable (Fig. 3c). Background ions were either H^+^ or K^+,^ and can be regarded as a measure of hydrolysed ATP (for further details, see Notes S2).

Also, in the compared scenario, the plant had to provide more C for one P molecule with increasing H/C conductance. However, the amount of released background ions (equivalent to hydrolysed ATP) decreased in a comparable manner (Fig. 3e). Thus, the transport of a positive charge (H/C) instead of no charge transport (SWEET) was favourable with respect to ATP consumption. If, however, C and energy metabolism were not limiting factors for the plant, this energetic disadvantage was overcompensated by the advantage of higher P transfer.

In another example of the fifth ‘*what‐if*’ case of electroneutral nutrient release, we considered the transport of an N source via ammonia permeases. Diffusion facilitators of this type recruit NH_4_^+^, catalyse the deprotonation to NH_3_ and allow the passage of this uncharged molecule to the other membrane side, where it regains its proton from the solution of the new compartment, with the proton from the deprotonation process remaining in the original compartment (Guether *et al*., 2009). Thus, there is no charge transport across the membrane. The driving force for NH_3_ transport through ammonia permeases was provided by the NH_3_ gradient, which was strongly dependent on the pH difference across the membrane. Because the (de)protonation reaction NH_4_^+^ ⇄ NH_3_ has a p*K*a ≈ 9.25, the free NH_3_ concentration dropped in acidic conditions, which, in turn, created a strong driving force from the less acidic to the more acidic compartment. Such an acid‐trapping mechanism (Wood *et al*., 2006) would be suitable for the release of ammonium from the fungus to the peri‐arbuscular space and would allow ammonium release even if the apoplastic ammonium concentration exceeded the cytosolic one (Fig. S3). This release, however, would be inert towards the P/C trade between plant and fungus, which means that the fungus would not benefit from the N source supply.

### Case 6: nutrient release together with a positive charge

**Figure 4:**
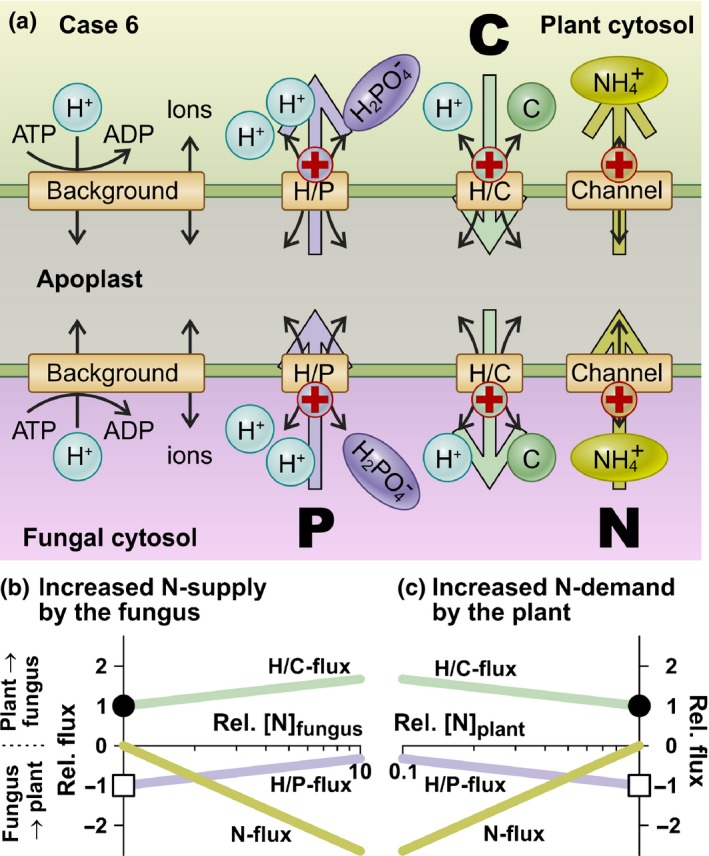
Hypothetical scenario for the efflux of nutrients together with a positive charge. (a) Nitrogen (N) could be shuttled across the fungal plasma membrane together with the transport of a positive charge as NH_4_^+^ via ammonium permeases (channel) and then transported across the plant plasma membrane together with a positive charge. The large coloured arrows indicate the prevalent fluxes in arbuscular mycorrhizal (AM) symbiosis. A ‘+’ indicates that transport of the nutrient is accompanied by the transport of a positive charge across the membrane. (b) Dependence of the carbon (C) (green, H/C flux), phosphorus (P) (purple, H/P flux) and N (yellow, N flux) fluxes on an increase in the relative concentration of the transported N source in the fungal cytosol. (c) Dependence of the C (green, H/C flux), P (purple, H/P flux) and N (yellow, N flux) fluxes on a decrease in the relative concentration of the transported N source in the plant cytosol. In the reference condition (rel. [N]_fungus_ = 1, black dots and white squares), the concentrations of the N source are identical in the plant and in the fungus (e.g. [N] = 0.5 mM). In the simulation examples shown, [P]_fungus_ : [P]_plant_ = 2 and [C]_plant_ : [C]_fungus_ = 2.

If we now consider the release of NH_4_^+^ as a positively charged ion, the fungus could harvest the battery effect (Gajdanowicz *et al*., 2011; Sandmann *et al*., 2011; Notes S2). Thus, from an energetic point of view with respect to the N/P/C trade, the release of charged ammonium via NH_4_^+^ channels has strong advantages for the fungus. Indeed, in AM symbiosis, NH_4_^+^ is thought to be the most common N form transferred from the fungus to the plant (Govindarajulu *et al*., 2005; Jin *et al*., 2005). Therefore, we enlarged the minimal model by one transporter type (NH_4_^+^ channel) in both membranes (Fig. 4). The conclusions, however, would also hold true for other N sources, such as peptides, amino acids or nitrate for instance, as long as they are transported together with a positive charge (e.g. via H/AA symporters or 2H/NO_3_^−^ symporters). We started the simulation of the network with equal concentrations of the transported N source in the plant and fungal cytosol (rel. [N]_plant_ = rel. [N]_fungus_ = 1). Under this condition, the enlarged network behaved in an identical manner to the minimal network without N transporter (Fig. 1). There was a constant flux of phosphate via the H/P symporters from the fungus to the plant (Fig. 4b,c, white squares), accompanied by a constant flow of the C source in the opposite direction (Fig. 4b,c, black dots). If the N concentration in the fungal cytosol was increased, there was an N flux from the fungus to the plant and the H/C flux was further stimulated, whereas the H/P flux decreased (Fig. 4b). The same phenomenon was observed if the N concentration in the plant was decreased (Fig. 4c). The fluxes did not depend on the absolute concentrations in the plant and the fungus, but only on the gradients between the organisms (Fig. S4a). If both partners had the same concentration of the N source, there was no N flux. More of the N source in the fungal cytosol (Fig. 4b) could be considered as a higher supply, whereas less of the N source in the plant (Fig. 4c) represented a higher demand.

It was noteworthy that an increase in the N flux was accompanied by a decrease in the P flux, and vice versa (Fig. S4a,b). This opposite trend resulted from the electric coupling of the N and P transport processes. An increased N flux from the fungus to the plant was accompanied by a larger efflux of positive charges across the fungal membrane and a larger influx of positive charges across the plant membrane. These N‐associated charge fluxes weakened the electrical component of the driving forces for P transport at both membranes. A reciprocal effect was observable if the P flux was stimulated (Fig. S4b). In this case, the P‐associated charge fluxes exerted a negative influence on the electrical component of the driving forces for N transport.

In conclusion, the release of a nutrient together with the transport of a positive charge (Fig. 4) was beneficial for the releasing organism, because it partially relieved the dependence of nutrient uptake processes on the H^+^‐ATPase. With respect to the N exchange in AM symbiosis, the most straightforward N transfer is the electrogenic release of NH_4_^+^ by the fungus and the electrogenic uptake of NH_4_^+^ by the plant. However, in emergency cases, when the cytosolic ammonium concentration approaches toxic levels, the release of NH_3_ with subsequent acid trapping in the peri‐arbuscular space would also make sense for both organisms (Coskun *et al*., 2013; Fig. S3).

## Discussion

**Figure 5:**
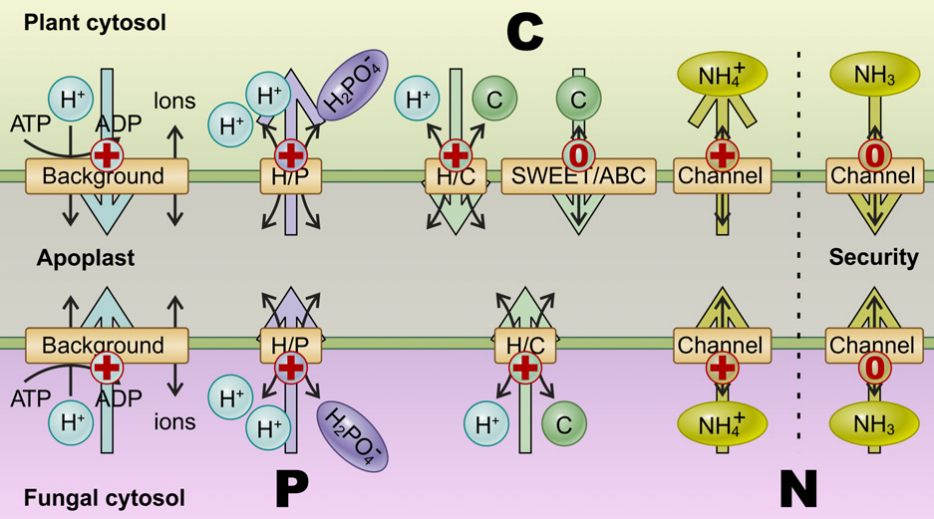
A thermodynamically optimized model for the nitrogen (N)/phosphorus (P)/carbon (C) exchange in arbuscular mycorrhizal (AM) symbiosis. In principle, the different nutrients are transported together with a positive charge across the membrane. However, in particular cases, they can also be shuttled in an electroneutral form. Ammonia channels can release NH_3_, SWEETs can release sugars and ABC transporters are putatively involved in exporting fatty acids (probably in an electroneutral way).

In this study, we have examined the nutrient exchange in AM symbiosis from a thermodynamic point of view and treated the problem without preconceptions. We played blind with regard to knowledge on the identity of transporter proteins, except that the uptake of the nutrients is energized by H^+^‐ATPases in both plant and fungus. As an outcome, we obtained a comprehensive view on the dynamic potential of nutrient exchange in AM symbiosis that rests on the grounds of first principle thermodynamics, and deduced a general transporter network for an optimal P/N/C exchange (Fig. 5). Interestingly, this general network looks similar to the minimal network proposed previously for the P/C trade (Schott *et al*., 2016). Indeed, if we model P and N as one combined commodity, the results presented previously for the P/C exchange would be (from a technical/mathematical/computational point of view) identical for a (PN)/C trade. Thinking one step further, even more nutrients could be accommodated in the different commodities without losing the principle dynamic features. In this context, it should be noted that the physiological interpretation of the simulation results becomes more complex as more entities are considered. Of course, the fate of the different nutrients (e.g. P and N) in subsequent metabolism is very different. However, before being metabolized, all nutrients need to pass the bottleneck of the two‐membrane system at the plant–fungus interface, and there they share the same transport restrictions elaborated in this study.

### Features of the transporter network fit to plant metabolism

The question ‘How harmonious are AM symbioses?’ is still widely discussed (Smith & Smith, 2015), and each experiment approaches this topic from a different perspective. A reasonable and rather successful concept describes AM symbiosis as a biological market (Kiers *et al*., 2011; Hammerstein & Noë, 2016; Noë & Kiers, 2018). In this context, our model now explains the thermodynamic basis of the underlying market forces. If we consider the transporter network for each partner separately, each subnetwork would perfectly serve for P, N and C accumulation (except SWEET). Thus, in principle, plant and fungus might be considered to compete with each other for the same resources, in particular P and N. This competitive predisposition, together with the different availability of the traded goods (the plant has better access to C, the fungus has better access to N and P), has self‐organizing features and stabilizes a robust cooperation. Apparently, these features are further amplified by the metabolism of the plant. Both N and phosphate starvation hamper amino acid and nucleic acid metabolism, and result in increased sucrose levels (Weber *et al*., 1998; Rouached *et al*., 2010). An increased sucrose level in cells colonized with AM fungi in turn stimulates the nutrient exchange and results in an increased P and N transfer to the plant (Fig. S4c,d). Thus, the features of the predicted optimal transporter network fit to general plant metabolism.

### Conclusions from the models

It is important to note that the models presented here assume that neither plant nor fungus exhibits an altruistic behaviour. Instead, we postulate that both organisms are only interested in maximizing their gains for reasonable costs. Our approach concludes with a transporter network (Fig. 5) which is not in conflict with those that were sketched according to experimentally verified transporter expression data (Parniske, 2008; Bonfante & Genre, 2010; Smith & Smith, 2011; Behie & Bidochka, 2014; Wang *et al*., 2017; Chen *et al*., 2018). This fact validates our assumption and, moreover, our approach sheds light on the question marks that were still assigned to several transport pathways. Compared with those networks already described elsewhere, our model is based on a solid thermodynamic foundation.

1. It is still unknown and widely discussed how the fungus releases phosphate. Our model indicates that anion channels would indeed have been well suited for the release of phosphate, if ‘release’ was the sole purpose from a fungal viewpoint. However, when it comes to obtaining another nutrient in return for phosphate, the modelled ‘*what‐if*’ scenarios provide strong arguments against the involvement of anion channels. Instead, they further substantiate the notion that phosphate is released by proton‐coupled phosphate transporters (Schott *et al*., 2016), which so far have been associated with phosphate uptake only. Meanwhile, this conclusion is also supported by experimental data. The proton‐coupled phosphate transporter GigmPT from the fungus *Gigaspora margarita* has been shown to be required for AM symbiosis (Xie *et al*., 2016). The finding that the silencing of GigmPT hampers the development of the fungus during symbiosis can be explained by a nutrient trade barrier, if the fungus cuts the phosphate supply (Schott *et al*., 2016). Likewise, the proton‐coupled phosphate transporter HcPT2 from the fungus *Hebeloma cylindrosporum* has been shown recently to be important for the unloading of radiolabelled ^32^Pi towards *Pinus pinaster* roots during this ectomycorrhizal association (Becquer *et al*., 2018).
2. The model predicts the existence of nutrient status‐dependent regulations of the different transporters. For example, it has been proposed previously that the plant sugar transporters should be regulated in response to the plant's intracellular phosphate level (Schott *et al*., 2016). Indeed, different experimental evidence points to diverse nutrient status‐dependent regulation cascades (Roth & Paszkowski, 2017; Wang *et al*., 2017).
3. Our analyses indicate that the release of C sources in an electroneutral manner, albeit not optimal in terms of energetics and C use efficiency, could be beneficial in terms of P acquisition. Interestingly, SWEETs involved in the export of sugars, and homologs of adenosine triphosphate‐binding cassette (ABC) transporters, putatively involved in the export of fatty acids, have been found to be essential for, or upregulated during, arbuscule formation (Zhang *et al*., 2010; Gutjahr *et al*., 2012; Manck‐Götzenberger & Requena, 2016). Such an upregulation of SWEET expression is not uncommon in plant–microbe interactions on infection (Bezrutczyk *et al*., 2018). Although the physiological reason for this reaction is still unclear, the upregulation of a set of C transporters might be considered as an evolutionary relict of plant defence reactions against intruders. In AM symbiosis, the sophisticated dynamics of the plant–fungus transporter network might have converted the initial defence reaction into a nutrient trade.

### From symbiosis to parasitism

A plant–fungus interaction does not always end up in a symbiotic partnership. Instead, while the same plant–fungus interaction may result in a symbiosis in one environmental condition, it may lead to a parasitic infection in another condition (Eaton *et al*., 2011; Nouri *et al*., 2014). Our models do not claim to comprehensively explain all aspects of this dualism. However, they could provide some further insights. As argued in Schott *et al*. (2016) and also here, AM symbiosis might be considered as the result of two organisms competing for the same nutrient resources (mainly phosphate and N). The main driving force of nutrient uptake is provided by the activity of the plasma membrane H^+^‐ATPases. Schott *et al*. (2016) have simulated modifications in this parameter and concluded that a mutual nutrient trade establishes only if both partners are equal in proton pumping activity, whereas partners with lower activity will be exploited.

The initial modelling approach of AM symbiosis was also applied to the infection of *Zea mays* by the parasitic fungus *Ustilago maydis* (Wittek *et al*., 2017). If the fungus reaches the phloem, a tissue with an extremely high sugar concentration, the symbiotic mechanism no longer works. The sugar gradient from the plant to the fungus cannot be compensated by any ‘membrane‐biophysical effort’ of the plant (e.g. increasing the pump activity). If the plant does not shield the infected tissue (e.g. by necrosis), it is doomed to nourish the fungus without any compensation.

### Upscaling of the model – from a single AM symbiosis to a complex symbiotic network

Usually, individual roots can be colonized by many AM fungal taxa (Smith & Smith, 2011) and not only by one as in the presented models. Interestingly, although elaborated for one plant–fungus pair only, the model for nutrient exchange is not restricted to this simplified case. It could easily be extended to more complex symbiotic interactions. In this case, the model of Fig. 5 must be considered as one module describing the interaction between two partners. For the description of a network of further partners, it is necessary to link many such individual modules in a larger network. Each of these modules would be governed by its own set of parameters. The different modules would share some of the parameters (e.g. concentrations or membrane voltages), which would result in several levels of coupling between them. An analysis of these more complex, higher order models goes beyond the scope of this article. In principle, however, it would be possible to also explain the transfer of nutrients from one fungus via the plant as intermediary to another fungus; or, if different hyphae of the same fungus colonize different plants, nutrient transfer between these plants might also be possible. The model (Fig. 5) also provides a fundamental basis for these scenarios.

It should also be noted that the considerations and conclusions presented here are not limited to the case of AM symbiosis. In principle, they are – as a whole or in part – applicable to any system in which two compartments/cells/organisms are separated by two adjacent membranes. Thus, the thermodynamic models can easily be adapted to parasitic fungus–plant interactions (Wittek *et al*., 2017) or generalized to other forms of mycorrhiza (Behie & Bidochka, 2014; Garcia *et al*., 2016; Field & Pressel, 2018; Nehls & Plassard, 2018).

### Outlook

Although the modelling approach presented in this study provides a comprehensive, thermodynamically funded view on the dynamics of nutrient exchange at the membrane interface in AM symbiosis, it should be considered as a starting point. It opens the door for future studies, in which wet‐laboratory experiments can be combined with computational simulations to obtain a deeper understanding of the investigated phenomena to further improve and validate the modelling approach.

## Author contributions

Conceptualization: ID. Investigation: ID, OS, KK, KM, MH, SA, SS‐V, CN‐R, MER‐M, JLG‐P, JR, AS, AZ. Writing – original draft: ID. Writing – review & editing: all authors. Supervision: ID, MAM‐M, AZ, SBG, PB, LS, HG. Funding acquisition: ID, MAM‐M, SBG, PB, LS, HG. OS, KK, KM, MH and SA contributed equally to this work.

## Supporting information

Please note: Wiley Blackwell are not responsible for the content or functionality of any Supporting Information supplied by the authors. Any queries (other than missing material) should be directed to the *New Phytologist* Central Office.

**Fig. S1** Additional explanations to Fig. 2(d–f).
**Fig. S2** Additional sugar efflux from the plant via diffusion facilitators.
**Fig. S3** Equilibrium conditions of ammonium transfer between fungus and plant in different scenarios.
**Fig. S4** Dependence of the nitrogen (N), phosphorus (P) and carbon (C) fluxes on the concentration gradients between plant and fungus.
**Methods S1** Further details on the modelling approach.
**Notes S1** Considerations towards the release of carbon (C) in the form of organic acids.
**Notes S2** The influence of ion transport on the background conductance – the K^+^ battery.

## References

[nph15646-bib-0001] Becquer A , Garcia K , Amenc L , Rivard C , Doré J , Trives‐Segura C , Szponarski W , Russet S , Baeza Y , Lassalle‐Kaiser B *et al* 2018 The *Hebeloma cylindrosporum* HcPT2 Pi transporter plays a key role in ectomycorrhizal symbiosis. New Phytologist 220: 1185–1199. 29944179 10.1111/nph.15281

[nph15646-bib-0002] Behie SW , Bidochka MJ . 2014 Nutrient transfer in plant–fungal symbioses. Trends in Plant Science 19: 734–740. 25022353 10.1016/j.tplants.2014.06.007

[nph15646-bib-0003] Bezrutczyk M , Yang J , Eom J‐S , Prior M , Sosso D , Hartwig T , Szurek B , Oliva R , Vera‐Cruz C , White FF *et al* 2018 Sugar flux and signaling in plant–microbe interactions. The Plant Journal 93: 675–685. 29160592 10.1111/tpj.13775

[nph15646-bib-0004] Bonfante P , Genre A . 2010 Mechanisms underlying beneficial plant–fungus interactions in mycorrhizal symbiosis. Nature Communications 1: 48. 10.1038/ncomms1046 20975705

[nph15646-bib-0005] Bravo A , Brands M , Wewer V , Dörmann P , Harrison MJ . 2017 Arbuscular mycorrhiza‐specific enzymes FatM and RAM2 fine‐tune lipid biosynthesis to promote development of arbuscular mycorrhiza. New Phytologist 214: 1631–1645. 28380681 10.1111/nph.14533

[nph15646-bib-0006] Bravo A , York T , Pumplin N , Mueller LA , Harrison MJ . 2016 Genes conserved for arbuscular mycorrhizal symbiosis identified through phylogenomics. Nature Plants 2: 15208. 27249190 10.1038/nplants.2015.208

[nph15646-bib-0007] Britto DT , Kronzucker HJ . 2006 Futile cycling at the plasma membrane: a hallmark of low‐affinity nutrient transport. Trends in Plant Science 11: 529–534. 17035071 10.1016/j.tplants.2006.09.011

[nph15646-bib-0008] Chen A , Gu M , Wang S , Chen J . 2018 Transport properties and regulatory roles of nitrogen in arbuscular mycorrhizal symbiosis. Seminars in Cell & Developmental Biology 74: 80–88. 28647533 10.1016/j.semcdb.2017.06.015

[nph15646-bib-0009] Chen L‐Q , Hou B‐H , Lalonde S , Takanaga H , Hartung ML , Qu X‐Q , Guo W‐J , Kim J‐G , Underwood W , Chaudhuri B *et al* 2010 Sugar transporters for intercellular exchange and nutrition of pathogens. Nature 468: 527–532. 21107422 10.1038/nature09606 PMC3000469

[nph15646-bib-0010] Coskun D , Britto DT , Li M , Becker A , Kronzucker HJ . 2013 Rapid ammonia gas transport accounts for futile transmembrane cycling under NH_3_/NH_4_ ^+^ toxicity in plant roots. Plant Physiology 163: 1859–1867. 24134887 10.1104/pp.113.225961 PMC3850193

[nph15646-bib-0011] Dreyer I . 2017 Plant potassium channels are in general dual affinity uptake systems. AIMS Biophysics 4: 90–106.

[nph15646-bib-0012] Dreyer I , Gomez‐Porras JL , Riaño‐Pachón DM , Hedrich R , Geiger D . 2012 Molecular evolution of slow and quick anion channels (SLACs and QUACs/ALMTs). Frontiers in Plant Science 3: 263. 23226151 10.3389/fpls.2012.00263 PMC3509319

[nph15646-bib-0013] Dreyer I , Gomez‐Porras JL , Riedelsberger J . 2017 The potassium battery: a mobile energy source for transport processes in plant vascular tissues. New Phytologist 216: 1049–1053. 28643868 10.1111/nph.14667

[nph15646-bib-0014] Eaton CJ , Cox MP , Scott B . 2011 What triggers grass endophytes to switch from mutualism to pathogenism? Plant Science 180: 190–195. 21421360 10.1016/j.plantsci.2010.10.002

[nph15646-bib-0015] Field KJ , Pressel S . 2018 Unity in diversity: structural and functional insights into the ancient partnerships between plants and fungi. New Phytologist 220: 996–1011. 29696662 10.1111/nph.15158

[nph15646-bib-0016] Finlay RD . 2008 Ecological aspects of mycorrhizal symbiosis: with special emphasis on the functional diversity of interactions involving the extraradical mycelium. Journal of Experimental Botany 59: 1115–1126. 18349054 10.1093/jxb/ern059

[nph15646-bib-0017] Gajdanowicz P , Michard E , Sandmann M , Rocha M , Corrêa LGG , Ramírez‐Aguilar SJ , Gomez‐Porras JL , González W , Thibaud J‐B , van Dongen JT *et al* 2011 Potassium (K^+^) gradients serve as a mobile energy source in plant vascular tissues. Proceedings of the National Academy of Sciences, USA 108: 864–869. 10.1073/pnas.1009777108 PMC3021027 21187374

[nph15646-bib-0018] Garcia K , Doidy J , Zimmermann SD , Wipf D , Courty P‐E . 2016 Take a trip through the plant and fungal transportome of mycorrhiza. Trends in Plant Science 21: 937–950. 27514454 10.1016/j.tplants.2016.07.010

[nph15646-bib-0019] Govindarajulu M , Pfeffer PE , Jin H , Abubaker J , Douds DD , Allen JW , Bücking H , Lammers PJ , Shachar‐Hill Y . 2005 Nitrogen transfer in the arbuscular mycorrhizal symbiosis. Nature 435: 819–823. 15944705 10.1038/nature03610

[nph15646-bib-0020] Guether M , Neuhäuser B , Balestrini R , Dynowski M , Ludewig U , Bonfante P . 2009 A mycorrhizal‐specific ammonium transporter from *Lotus japonicus* acquires nitrogen released by arbuscular mycorrhizal fungi. Plant Physiology 150: 73–83. 19329566 10.1104/pp.109.136390 PMC2675747

[nph15646-bib-0021] Gutjahr C , Radovanovic D , Geoffroy J , Zhang Q , Siegler H , Chiapello M , Casieri L , An K , An G , Guiderdoni E *et al* 2012 The half‐size ABC transporters STR1 and STR2 are indispensable for mycorrhizal arbuscule formation in rice. The Plant Journal 69: 906–920. 22077667 10.1111/j.1365-313X.2011.04842.x

[nph15646-bib-0022] Hammerstein P , Noë R . 2016 Biological trade and markets. Philosophical Transactions of the Royal Society of London. Series B: Biological Sciences 371: 2015.0101. 10.1098/rstb.2015.0101 PMC4760201 26729940

[nph15646-bib-0023] Hodge A , Stewart J , Robinson D , Griffiths BS , Fitter AH . 2000 Competition between roots and soil micro‐organisms for nutrients from nitrogen‐rich patches of varying complexity. Journal of Ecology 88: 150–164.

[nph15646-bib-0024] Jacoby R , Peukert M , Succurro A , Koprivova A , Kopriva S . 2017 The role of soil microorganisms in plant mineral nutrition—current knowledge and future directions. Frontiers in Plant Science 8: 1617. 28974956 10.3389/fpls.2017.01617 PMC5610682

[nph15646-bib-0025] Jiang Y , Wang W , Xie Q , Liu N , Liu L , Wang D , Zhang X , Yang C , Chen X , Tang D *et al* 2017 Plants transfer lipids to sustain colonization by mutualistic mycorrhizal and parasitic fungi. Science (New York, N.Y.) 356: 1172–1175. 10.1126/science.aam9970 28596307

[nph15646-bib-0026] Jin H , Pfeffer PE , Douds DD , Piotrowski E , Lammers PJ , Shachar‐Hill Y . 2005 The uptake, metabolism, transport and transfer of nitrogen in an arbuscular mycorrhizal symbiosis. New Phytologist 168: 687–696. 16313650 10.1111/j.1469-8137.2005.01536.x

[nph15646-bib-0027] Keymer A , Pimprikar P , Wewer V , Huber C , Brands M , Bucerius SL , Delaux P‐M , Klingl V , von Röpenack‐Lahaye E , Wang TL *et al* 2017 Lipid transfer from plants to arbuscular mycorrhiza fungi. eLife 6: e29107. 10.7554/eLife.29107 PMC5559270 28726631

[nph15646-bib-0028] Kiers ET , Duhamel M , Beesetty Y , Mensah JA , Franken O , Verbruggen E , Fellbaum CR , Kowalchuk GA , Hart MM , Bago A *et al* 2011 Reciprocal rewards stabilize cooperation in the mycorrhizal symbiosis. Science 333: 880–882. 21836016 10.1126/science.1208473

[nph15646-bib-0029] Kim SJ , Eo J‐K , Lee E‐H , Park H , Eom A‐H . 2017 Effects of arbuscular mycorrhizal fungi and soil conditions on crop plant growth. Mycobiology 45: 20. 28435350 10.5941/MYCO.2017.45.1.20 PMC5395496

[nph15646-bib-0030] Lanfranco L , Bonfante P , Genre A . 2016 The mutualistic interaction between plants and arbuscular mycorrhizal fungi. Microbiology Spectrum 4: 727–747. 10.1128/microbiolspec.FUNK-0012-2016 28087942

[nph15646-bib-0031] Li N , Xu C , Li‐Beisson Y , Philippar K . 2016 Fatty acid and lipid transport in plant cells. Trends in Plant Science 21: 145–158. 26616197 10.1016/j.tplants.2015.10.011

[nph15646-bib-0032] Loew LM , Schaff JC . 2001 The Virtual Cell: a software environment for computational cell biology. Trends in Biotechnology 19: 401–406. 11587765 10.1016/S0167-7799(01)01740-1

[nph15646-bib-0033] Luginbuehl LH , Menard GN , Kurup S , Van Erp H , Radhakrishnan GV , Breakspear A , Oldroyd GED , Eastmond PJ . 2017 Fatty acids in arbuscular mycorrhizal fungi are synthesized by the host plant. Science 356: 1175–1178. 28596311 10.1126/science.aan0081

[nph15646-bib-0034] MacLean AM , Bravo A , Harrison MJ . 2017 Plant signaling and metabolic pathways enabling arbuscular mycorrhizal symbiosis. Plant Cell 29: 2319–2335. 28855333 10.1105/tpc.17.00555 PMC5940448

[nph15646-bib-0035] Manck‐Götzenberger J , Requena N . 2016 Arbuscular mycorrhiza symbiosis induces a major transcriptional reprogramming of the potato SWEET sugar transporter family. Frontiers in Plant Science 7: 487. 27148312 10.3389/fpls.2016.00487 PMC4830831

[nph15646-bib-0036] Nehls U , Plassard C . 2018 Nitrogen and phosphate metabolism in ectomycorrhizas. New Phytologist 220: 1047–1058. 29888395 10.1111/nph.15257

[nph15646-bib-0037] Noë R , Kiers ET . 2018 Mycorrhizal markets, firms, and co‐ops. Trends in Ecology & Evolution 33: 777–789. 30177306 10.1016/j.tree.2018.07.007

[nph15646-bib-0038] Nouri E , Breuillin‐Sessoms F , Feller U , Reinhardt D . 2014 Phosphorus and nitrogen regulate arbuscular mycorrhizal symbiosis in *Petunia hybrida* . PLoS ONE 9: e90841. 24608923 10.1371/journal.pone.0090841 PMC3946601

[nph15646-bib-0039] Parniske M . 2008 Arbuscular mycorrhiza: the mother of plant root endosymbioses. Nature Reviews. Microbiology 6: 763–775. 18794914 10.1038/nrmicro1987

[nph15646-bib-0040] Roth R , Paszkowski U . 2017 Plant carbon nourishment of arbuscular mycorrhizal fungi. Current Opinion in Plant Biology 39: 50–56. 28601651 10.1016/j.pbi.2017.05.008

[nph15646-bib-0041] Rouached H , Arpat AB , Poirier Y . 2010 Regulation of phosphate starvation responses in plants: signaling players and cross‐talks. Molecular Plant 3: 288–299. 20142416 10.1093/mp/ssp120

[nph15646-bib-0042] Sandmann M , Skłodowski K , Gajdanowicz P , Michard E , Rocha M , Gomez‐Porras JL , González W , Corrêa LGG , Ramírez‐Aguilar SJ , Cuin TA *et al* 2011 The K^+^ battery‐regulating Arabidopsis K^+^ channel AKT2s under the control of multiple post‐translational steps. Plant Signaling and Behavior 6: 558–562. 21445013 10.4161/psb.6.4.14908 PMC3142392

[nph15646-bib-0043] Schott S , Valdebenito B , Bustos D , Gomez‐Porras JL , Sharma T , Dreyer I . 2016 Cooperation through competition – dynamics and microeconomics of a minimal nutrient trade system in arbuscular mycorrhizal symbiosis. Frontiers in Plant Science 7: 912. 27446142 10.3389/fpls.2016.00912 PMC4921476

[nph15646-bib-0044] Sharma T , Dreyer I , Kochian L , Piñeros MA . 2016 The ALMT family of organic acid transporters in plants and their involvement in detoxification and nutrient security. Frontiers in Plant Science 7: 1488. 27757118 10.3389/fpls.2016.01488 PMC5047901

[nph15646-bib-0045] Smith FA , Smith SE . 2015 How harmonious are arbuscular mycorrhizal symbioses? Inconsistent concepts reflect different mindsets as well as results. New Phytologist 205: 1381–1384. 25420770 10.1111/nph.13202

[nph15646-bib-0046] Smith SE , Read DJ . 2008 Mycorrhizal symbiosis. Cambridge, UK: Academic Press.

[nph15646-bib-0047] Smith SE , Smith FA . 2011 Roles of arbuscular mycorrhizas in plant nutrition and growth: new paradigms from cellular to ecosystem scales. Annual Review of Plant Biology 62: 227–250. 10.1146/annurev-arplant-042110-103846 21391813

[nph15646-bib-0048] Wang W , Shi J , Xie Q , Jiang Y , Yu N , Wang E . 2017 Nutrient exchange and regulation in arbuscular mycorrhizal symbiosis. Molecular Plant 10: 1147–1158. 28782719 10.1016/j.molp.2017.07.012

[nph15646-bib-0049] Weber H , Golombek S , Heim U , Borisjuk L , Panitz R , Manteuffel R , Wobus U . 1998 Integration of carbohydrate and nitrogen metabolism during legume seed development: implications for storage product synthesis. Journal of Plant Physiology 152: 641–648.

[nph15646-bib-0050] Wittek A , Dreyer I , Al‐Rasheid KAS , Sauer N , Hedrich R , Geiger D . 2017 The fungal UmSrt1 and maize ZmSUT1 sucrose transporters battle for plant sugar resources. Journal of Integrative Plant Biology 59: 422–435. 28296205 10.1111/jipb.12535

[nph15646-bib-0051] Wood CC , Porée F , Dreyer I , Koehler GJ , Udvardi MK . 2006 Mechanisms of ammonium transport, accumulation, and retention in oocytes and yeast cells expressing Arabidopsis AtAMT1;1. FEBS Letters 580: 3931–3936. 16806203 10.1016/j.febslet.2006.06.026

[nph15646-bib-0052] Xie X , Lin H , Peng X , Xu C , Sun Z , Jiang K , Huang A , Wu X , Tang N , Salvioli A *et al* 2016 Arbuscular mycorrhizal symbiosis requires a phosphate transceptor in the *Gigaspora margarita* fungal symbiont. Molecular Plant 9: 1583–1608. 27688206 10.1016/j.molp.2016.08.011

[nph15646-bib-0053] Zhang Q , Blaylock LA , Harrison MJ . 2010 Two *Medicago truncatula* half‐ABC transporters are essential for arbuscule development in arbuscular mycorrhizal symbiosis. Plant Cell 22: 1483–1497. 20453115 10.1105/tpc.110.074955 PMC2899874

