# Supplementary material for: Nutrient exchange in arbuscular mycorrhizal symbiosis from a thermodynamic point of view
Source: New Phytol. 2019 Jan 21;222(2):1043–53. doi: 10.1111/nph.15646 (PMC6667911; doi:10.1111/nph.15646)
Supplement: Supplementary file 1 — Fig. S1 Additional explanations to Fig. 2(d–f). Fig. S2 Additional sugar efflux from the plant via diffusion facilitators. Fig. S3 Equilibrium conditions of ammonium transfer between fungus and plant in different scenarios. Fig. S4 Dependence of the nitrogen (N), phosphorus (P) and carbon (C) fluxes on the concentration gradients between plant and fungus. Methods S1 Further details on the modelling approach. Notes S1 Considerations towards the release of carbon (C) in the form of organic acids. Notes S2 The influence of ion transport on the background conductance – the K+ battery. [file NPH-222-1043-s001.pdf]

New Phytologist **Supporting Information**

Article title:

Nutrient exchange in arbuscular mycorrhizal symbiosis from a thermodynamic point of view

Authors:
Ingo Dreyer, Olivia Spitz, Kerstin Kanonenberg, Karolin Montag, Maria Handrich, Sabahuddin Ahmad, Stephan Schott-Verdugo, Carlos Navarro-Retamal, María E. Rubio-Meléndez, Judith L. Gomez-Porras, Janin Riedelsberger, Marco A. Molina-Montenegro, Antonella Succurro, Alga Zuccaro, Sven B. Gould, Petra Bauer, Lutz Schmitt, Holger Gohlke

Article acceptance date: 07 December 2018

The following Supporting Information is available for this article:

Fig. S1 Additional explanations to Fig. 2d-f.

Fig. S2 Additional sugar efflux from the plant via diffusion facilitators.

Fig. S3 Equilibrium conditions of ammonium transfer between fungus and plant in different scenarios.

Fig. S4 Dependency of the N-, P-, and C-fluxes on the concentration gradients between plant and fungus.

Methods S1 Further details on the modelling approach.

Notes S1 Considerations towards the release of C in form of organic acids.

Notes S2 The influence of ion transport on the background conductance – the K^+ battery.

Figure S1

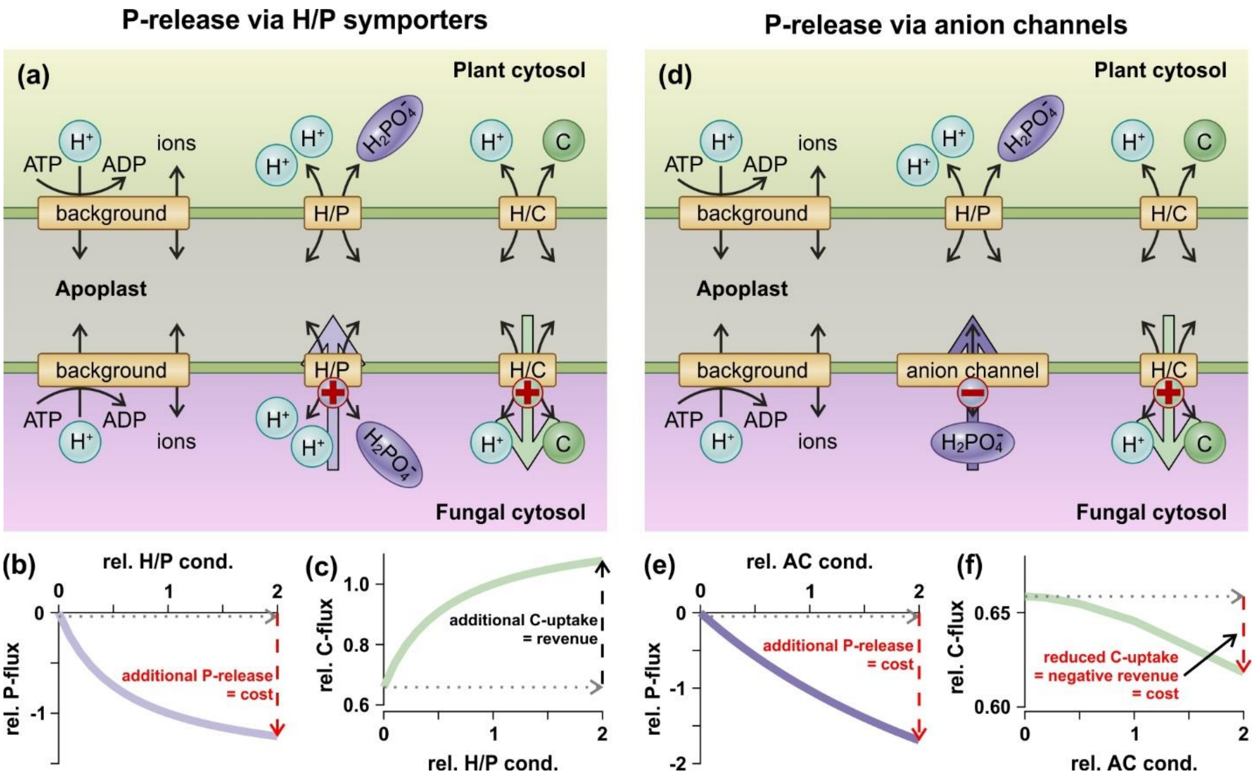

Fig. S1 Additional explanations to Fig. 2d-f. Two "what-if" scenarios are compared: in the left scenario (a-c) the fungus can release phosphate via proton-coupled symporters (H/P), while in the right scenario (d-f) it employs anion channels for this purpose. All other conditions are identical in both scenarios. In should be noted that the (qualitative) conclusions outlined in the following are not dependent on these environmental conditions and depend only on the different transport types of the two (putative) phosphate transporters. Solid curves in (b) and (c) show sections of Fig. 2f and solid curves in (e) and (f) show sections of Fig. 2e. Negative flux values = efflux from the fungus; positive values = nutrient uptake by the fungus. (b) When the H/P conductance is zero, there is no P-flux. If the relative conductance increases (e.g. to a value of 2; dotted arrow), the P-efflux increases (dashed arrow). In economic terms, this additional P-release is the cost of the conductance increase. (c) The H/P conductance-change (dotted arrow) also causes an increase of the C-influx (dashed arrow). In economic terms, this additional C-uptake is the revenue of the conductance increase. (e) As in panel (b) the increase of the anion channel conductance (dotted arrow) causes an additional P-release (dashed arrow, cost). (f) In contrast to (c) the increase of the anion channel conductance does not cause an increase but provokes a reduction in C-influx (dashed arrow). Thus, the "revenue" of the conductance-increase is negative and could be therefore considered as a cost.

The right scenario is not supported by experimental findings, while the left scenario is. It has not been observed experimentally that the fungus provides more phosphate and receives less carbon sources in return. In contrast, AM symbiosis is characterised by reciprocal rewards (Kiers *et al.*, 2011).

Nevertheless, in a thought experiment we can consider a hypothetical fungus that does not have H/P symporters, but is equipped only with phosphate-permeable anion channels. Because a fungus cannot be considered as an altruistic organism, this fungus would have to optimise its P / C exchange. In such an optimization process, the fungus would have to regulate the anion channel conductance so that it receives the most C for the least P. This condition would be: anion channel conductance = 0. Thus, even if the fungus had a phosphate-permeable anion channel it would not make any sense to use it in AM nutrient exchange.

Figure S2

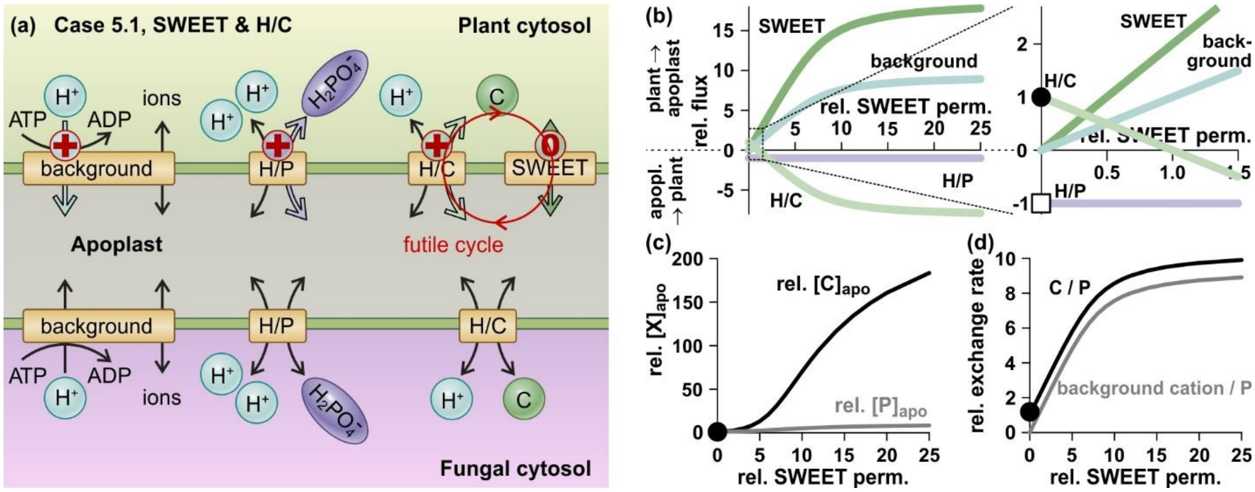

Fig. S2. Additional sugar efflux from the plant via diffusion facilitators. (a) The minimal model was enlarged by a sugar diffusion facilitator (SWEET). (b) SWEET proteins canalise the efflux of electroneutral C-sources very efficiently. As a consequence of an increased SWEET permeability, which could be achieved for instance by a higher expression rate, the C-efflux via SWEETs increases (dark green, SWEET), the C-efflux via the H/C-transporter decreases and converts its direction (light green, H/C), the P-flux via the H/P-transporter does not change remarkably (purple, H/P), and the flux mediated by the H^+-ATPase driven background conductance increases (blue, background). The panel on the right shows an enlargement of the boxed region on the left to illustrate better the reversal of the C-flux through H/C at a certain SWEET permeability. The SWEET permeability was normalised to 1 when the H/C-flux reverses direction. At this value and above, a futile cycle of C-efflux via SWEET and H^+-ATPase-driven C-influx via the H/C-transporter is established. The plant H/C-symporter is converted from an exporter into an importer due to the increase in the apoplastic C-concentration. (c) Dependency of apoplastic [C] (black) and [P] (grey) on the SWEET permeability. While $[C]_{apo}$ can increase by a factor >100, the raise of $[P]_{apo}$ is less pronounced. The concentrations were normalised to the condition in the absence of SWEETs: rel. SWEET perm. = 0,

rel. $[C]_{apo}$ = 1 and rel. $[P]_{apo}$ = 1. (d) The SWEET-induced decrease of the electrogenic H/C-efflux hardly affects the P-flux because the H^+-ATPase-dominated background conductance compensates for the altered charge movement. As a consequence, the plant does not only provide more C but has to release also cations via the background conductance. These might be protons, directly pumped by using the energy from ATP hydrolysis or K^+ ions, which were taken up before using the energy from ATP hydrolysis. With increasing SWEET permeability, the relative exchange rates C/P (black) and background cation/P (grey) increase as well. The C/P-curve indicates the amount of C provided per P, and the background cation/P-curve indicates the amount of released background cations, which is equivalent to the amount of ATP hydrolysed, per P. Thus, the plant does not only provide more C, but additionally has to invest more ATP per P-molecule obtained. Nevertheless, it should be noted that the plant does not suffer from a reduced P-acquisition. This is in contrast to the futile cycle established by a combination of H/P, anion channel, and pump (**Fig. 2**), which resulted in a decreased C-uptake by the fungus.

Figure S3

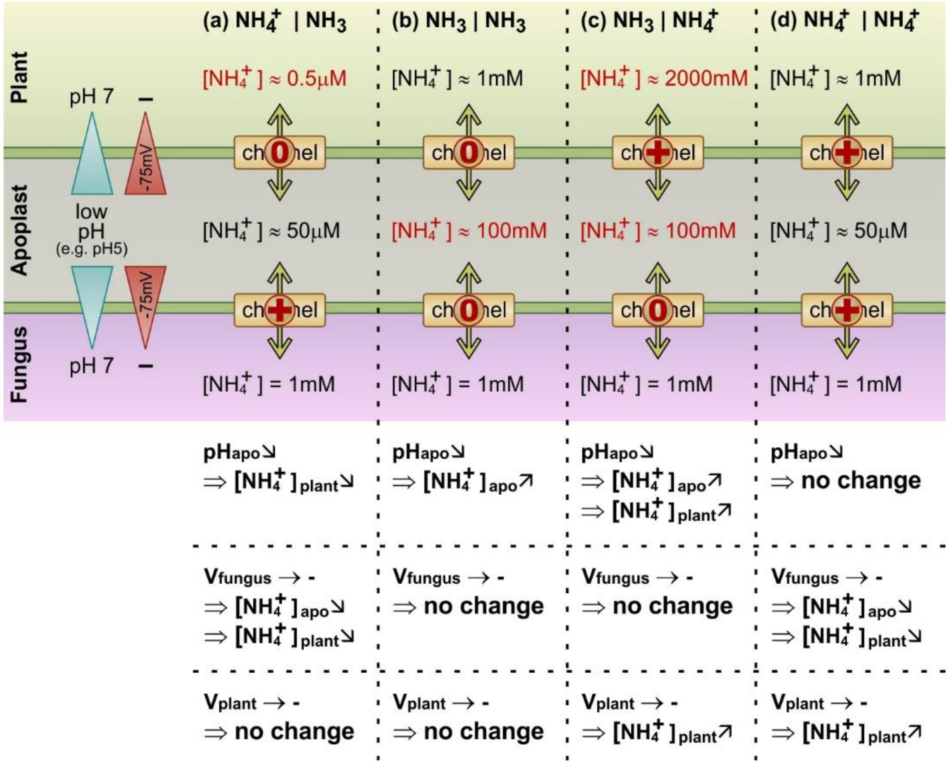

Fig. S3 Equilibrium conditions of ammonium transfer between fungus and plant in different scenarios. In principle, both plant and fungus could employ either ammonium (NH_4^+) or ammonia (NH_3) permeases for transmembrane ammonium transport. Both recruit NH_4^+. The first transporter type (NH_4^+ channel) allows

the passage of the molecule together with the positive charge across the membrane, while the second transporter type (NH_3 channel) catalyses the deprotonation of NH_4^+ to NH_3 and allows only the passage of this uncharged molecule to the other membrane side. At the other side NH_3 regains its proton from the solution of the new compartment while the proton from the deprotonation process remains in the original compartment (Guether *et al.*, 2009). Thus, there is no charge transport across the membrane.

Here, we considered all possible four combinations of the two transporter types (a-d) in thought experiments under an identical condition: In both organisms, the H^+-ATPase-driven background conductance establishes (i) a membrane voltage (e.g. -75mV) and (ii) a pH gradient (e.g. pH5 in the apoplast, while the cytosol has pH7). Exemplarily, for a cytosolic NH_4^+ concentration in the fungus $[NH_4^+]_{fungus}$ = 1mM the equilibrium concentrations $[NH_4^+]_{apoplast}$ and $[NH_4^+]_{plant}$ are calculated, at which there is no net flux across any membrane anymore. Based on these equilibrium values, plant and fungus can induce a net flux from the fungus to the plant if $[NH_4^+]_{plant}$ is reduced below the indicated value or if $[NH_4^+]_{fungus}$ is increased. Likewise, a net flux from the plant to the fungus can be induced if $[NH_4^+]_{plant}$ is increased or if $[NH_4^+]_{fungus}$ is reduced. (a) The fungus transports NH_4^+ while the plant transports NH_3. There is no net flux across either membrane if $[NH_4^+]_{apoplast}$ = 50μM and $[NH_4^+]_{plant}$ = 0.5μM. (b) Both fungus and plant transport NH_3. There is no net flux across either membrane if $[NH_4^+]_{apoplast}$ = 100mM and $[NH_4^+]_{plant}$ = 1mM. (c) The fungus transports NH_3 while the plant transports NH_4^+. There is no net flux across either membrane if $[NH_4^+]_{apoplast}$ = 100mM and $[NH_4^+]_{plant}$ = 2000mM. (d) Both fungus and plant transport NH_4^+. There is no net flux across either membrane if $[NH_4^+]_{apoplast}$ = 50μM and $[NH_4^+]_{plant}$ = 1mM.

The consequences of changes in the parameters pH_{apo}, V_{plant} (membrane voltage at the plant plasma membrane) and V_{fungus} (membrane voltage at the fungal plasma membrane) on the equilibrium concentrations of $[NH_4^+]_{plant}$, $[NH_4^+]_{apo}$, and $[NH_4^+]_{fungus}$ are presented in the lower panel: (a) The consequence of a lower $pH_{apoplast}$ would be an even lower equilibrium concentration for $[NH_4^+]_{plant}$. The consequences of a lower V_{fungus} would be lower equilibrium concentrations for $[NH_4^+]_{apoplast}$ and $[NH_4^+]_{plant}$. Changes in V_{plant} would not affect the equilibrium concentrations. (b) The consequence of a lower $pH_{apoplast}$ would be an even higher equilibrium concentration for $[NH_4^+]_{apoplast}$. Changes in V_{fungus} and V_{plant} would not affect the equilibrium concentrations. (c) The consequences of a lower $pH_{apoplast}$ would be even higher equilibrium concentrations for $[NH_4^+]_{apoplast}$ and $[NH_4^+]_{plant}$. Changes in V_{fungus} would not affect the equilibrium concentrations. The consequence of a lower V_{plant} would be an even higher equilibrium concentration for $[NH_4^+]_{plant}$. (d) A change in $pH_{apoplast}$ would not affect the equilibrium concentrations. The consequences of a lower V_{fungus} would be lower equilibrium concentrations for $[NH_4^+]_{apoplast}$ and $[NH_4^+]_{plant}$. The consequence of a lower V_{plant} would be a higher equilibrium concentration for $[NH_4^+]_{plant}$.

Panels (a) and (b) deal with the uptake of uncharged NH_3 by the plant. Interestingly, such an uptake process would not affect the charge-driven P/C-trade between plant and fungus; it does not consume any energy provided by the electrical or proton gradient. This apparent benefit, however, has a decisive disadvantage: under physiological conditions there is hardly sufficient energy for such an “inverse acid-trapping” transport from the peri-arbuscular space to the plant cytosol. Because the transmembrane

electrical gradient cannot be used, the transport needs to be driven exclusively by the chemical NH_3 gradient across the plant plasma membrane. This gradient, however, is compromised by the prevailing physiological conditions. The pH-dependent $NH_4^+ \rightarrow NH_3$ deprotonation process is energetically favoured at cytosolic pH7 compared to the lower pH in the peri-arbuscular space. Thus, there is a pH difference-dependent driving force from the cytosol to the apoplast that needs to be overcompensated by the chemical ammonium gradient. To achieve an electroneutral NH_3 uptake, the ammonium concentration in the peri-arbuscular space must be (i) more than 10-fold higher than the cytosolic one, if the pH difference is 1 (pH_{cyt}7.0, pH_{apo}6.0), (ii) more than 100-fold higher if the pH difference is 2 (pH_{cyt}7.0, pH_{apo}5.0; panels a,b), (iii) more than 1000-fold higher if the pH difference is 3 (pH_{cyt}7.0, pH_{apo}4.0). Theoretically, such gradients might be achieved if the plant could maintain sub-micromolar cytosolic ammonium concentrations (demand strategy, Schott *et al.*, 2016) or if the fungus floods the peri-arbuscular space with ammonium to concentrations of hundreds of millimolar (supply strategy). However, both scenarios are hard to realise and highly improbable. Moreover, the plant has little control over this type of transport process, because it strongly depends on the apoplastic pH, which is not influenced by the plant alone. Thus, thermodynamic constraints do not allow the electroneutral uptake of NH_3 by the plant in a robust manner.

Please note that the displayed scenarios are thought experiments. Concentrations displayed in red indicate values, which are questionable for physiologic conditions. Thus, the only physiologically meaningful scenarios are (d; without restrictions) and potentially (b) if cytosolic NH_4^+ concentrations reach toxic levels and need to be released urgently.

Figure S4

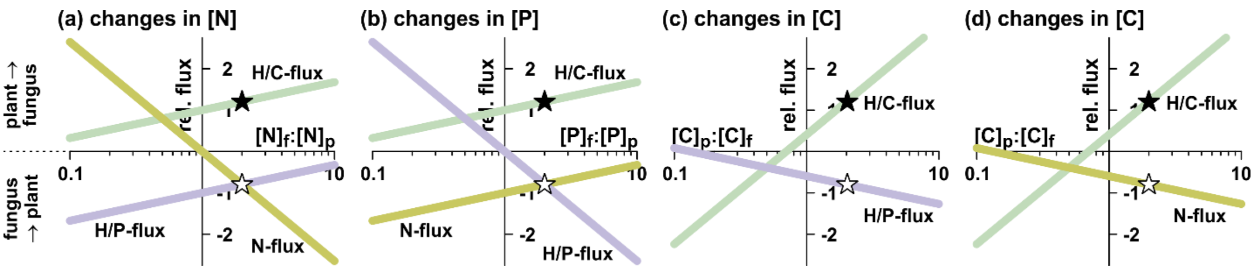

Fig. S4 Dependency of the N- (yellow, N-flux), P- (purple, H/P-flux), and C-fluxes (green, H/C-flux) on the concentration gradients between plant (p) and fungus (f). In the general model of **Fig. 5** the electroneutral transporters (SWEET/ABC and NH_3 channels) were silenced. The stars indicate the starting condition for all scenarios: $[N]_f:[N]_p = 2$; $[P]_f:[P]_p = 2$; $[C]_p:[C]_f = 2$. (a) Variation of the [N]-gradient, (b) variation of the [P]-gradient, (c,d) Variation of the [C]-gradient. Please note: (c) and (d) present P- and N-fluxes individually because in one graph the two curves would superimpose.

Methods S1 Further details on the modelling approach.

Voltages at the plant and fungal plasma membranes couple transport processes

For each plasma membrane, the respective membrane voltage was calculated independently as free-running membrane potential difference. Several of the transport processes considered in this study are electrogenic, *i.e.* the transport of a nutrient molecule is accompanied by the transport of a net charge. Such a charge transport changes strongly the voltage at the respective membrane, which in turn feeds back on the nutrient transport and results in a strong coupling of the otherwise not connected transport processes. When starting a simulation with randomly chosen start values of the free running parameters (concentrations in the apoplast and membrane voltages), they are not yet stationary but change. Concentrations change according to

$$\frac{\partial}{\partial t}[X] = J \cdot \frac{A}{Vol} \qquad \text{(eq. S1)}$$

and the membrane voltage changes according to

$$\frac{\partial}{\partial t}V = \frac{1}{C_M} \cdot I_A \qquad \text{(eq. S2)}$$

where J is the net flux density (unit $\frac{\mu mol}{\mu m^2 \cdot s}$), A is the membrane surface through which the flux flows, Vol is the volume of the target compartment, $C_M = 10^{-2}\,\frac{pF}{\mu m^2}$ is the specific membrane capacitance and I_A is the net current density (unit $\frac{pA}{\mu m^2}$). After a short equilibration phase (< 1s; see Schott *et al.*, 2016), all free-running parameters reach an equilibrium (*i.e.* $\frac{\partial}{\partial t}V = 0$ and $\frac{\partial}{\partial t}[X] = 0$), which depends on the setting of the other (pre-adjusted and screened) parameters. In this equilibrium phase, the net flux in and out of the periarbuscular space is zero ($J_{X\_plant \rightarrow apo} + J_{X\_fungus \rightarrow apo} = 0$). The different transmembrane fluxes, however, are usually different from zero and have system-dependent steady state levels ($J_{X\_plant \rightarrow apo} = -J_{X\_fungus \rightarrow apo}$). These are the physiologically important fluxes between plant and fungus. The constant value of the membrane voltage implies that there is no net charge transport across the membrane anymore. Thus, an electrogenic transport by one transporter must be compensated by an inverse electrogenic transport of another transporter, which in fact explains the coupling of otherwise unrelated transport processes.

Model assessment

Although each model is characterised by a large set of free or partially dependent parameters, it is always a simplified representation of the real situation. To avoid any oversimplification, we followed in our modelling approach the leitmotif: "*As simple as possible, but as complex as necessary!*" It should be emphasised that the necessary complexity might be partially hidden and not be evident at first sight. To gain confidence in the reliability of the simulations, the model approach was therefore carefully assessed.

(i) *Geometry.* The simulation results do not depend on the exact size of the interfacial apoplast between plant and fungus. The size of the periarbuscular space was set to 100 nm, a value reported in literature (Balestrini & Bonfante, 2005). This value was used to calculate the volume of the apoplast

between both membranes and to determine the concentration changes during the simulations. The increase or decrease of the distance between the membrane by a factor of 10 to 1 µm and 10 nm, respectively, affected in the simulation only the initial equilibration process by factor ~3 and ~0.8, respectively, but left the equilibrium conditions unaffected. The physiologically important fluxes between plant and fungus are those at equilibrium; they are independent of the exact geometry.

(ii) *Concentrations of H^+, P and C.* Under the conditions (i) $pH_{apo} < pH_{cyt}$, and (ii) $[P]_{plant} < [P]_{fungus}$, and (iii) $[C]_{fungus} < [C]_{plant}$, the presented simulation results do not depend on the exact values of the concentrations. For practical purposes, the pH values were set to physiological pH7.0 in the cytosols and to pH6.0 in the apoplast. Different relations, which obey the condition $pH_{apo} < pH_{cyt}$ do not affect qualitatively the results, although quantitatively they would do. A lower pH_{apo} (Guttenberger, 2000) would result in lower nutrient concentrations in the periarbuscular space, which would feed back on the concentration gradients and would increase the driving force of the considered transport processes. In a model, however, an increased driving force is redundant with an increase in other parameters (*e.g.* the channel conductance). Similar considerations apply to $[P]_{plant}$, $[P]_{fungus}$, $[C]_{plant}$, $[C]_{fungus}$. To eliminate this redundancy, to avoid unnecessary levels of complexity and to bypass the missing information on the exact pH- and concentration values, we pre-set the pH-values and the cytosolic concentrations of the nutrients ($[C]_{fungus}$ = 1mM < $[C]_{plant}$ = 2mM; $[P]_{fungus}$ = 3mM > $[P]_{plant}$ = 1.5mM) and kept them for all the simulations unless otherwise stated. Other values, which obey the above-mentioned conditions would produce the same qualitative results.

(iii) *Rescaling to relative fluxes.* It should be noted that we do not present absolute flux values, but relative flux changes in comparison to a standard condition. This is not a limitation of the modelling approach, but on the contrary a generalization. Herby, the described effects are not limited to a certain narrow parameter set, but can be assigned to a wide parameter range (for instance, see the comment on concentrations just before). As a consequence, we do neither provide absolute values for the model parameters but present relative changes, instead.

(iv) *Transporter-specific parameters.* Although not directly intuitive, the very simple representation of these parameters in the models covers a wide range of complexity. The composite parameters "maximum current", "conductance" or "permeability" (I_{max}, $G_{H/X}$, $Diff_{SWEET}$, $G_{NH_4^+}$, D_{NH_3}, G_{A^-}) usually depend in a complex manner on a variety of environmental parameters. Their value may change with the substrate concentration (Km-value) and may show saturation, or they may be regulated by the membrane voltage, by gene expression and/or downstream signalling cascades. Despite this hardly conceivable complexity, in the model they are described just by a simple value. It may not be immediately obvious that this simplification still covers all the outlined complexity and much more, but it does. As outlined before, we consider the (relative) fluxes in equilibrium conditions. In equilibrium all the parameters (also the free running parameters) have a fixed value and thus also the composite parameters have a fixed value. If we screen now these values in the entire reasonable interval (from 0 to ∞), we cover each potential regulatory effect without the need to specify it further.

The normalization of these values to the value in a reference condition simplifies the presentation (values in the reference condition are 1 in most cases) without restricting the generality of the results. The obtained data can then be interpreted in several different ways in the context of the specific scenario that is considered. For instance, the relative increase of a flux with an increasing conductance value can be interpreted as (i) the flux increases with higher transporter expression or as (ii) the flux increases with post-translational transporter activation. Both scenarios are represented by the same modelling result.

Taken together, these and additional considerations presented in Schott *et al.* (2016) provide the proper ground of a reliable modelling approach that removes redundancies in the parameters without losing complexity. Such an approach is the only viable one from a mathematical / computational perspective to capture this complex system. But admittedly, it requires a certain amount of abstraction. We kindly ask the readers for their comprehension.

Notes S1 Regarding the potential release of C in form of organic acids.

In the main text we conclude that for plants it does not make sense to release carbon sources in form of negatively charged organic acids via anion channels of the ALMT-type. One might argue now that plant roots do release larger amounts of organic acids via ALMTs (Sharma *et al.*, 2016). How does this fit with our conclusion that anion channels are not suited for AM nutrient exchange? The answer is that the nutrient release in large quantities by roots has a different purpose and occurs in a system with a different geometry. Therefore, the conditions in the peri-arbuscular space are not comparable to those in the open rhizosphere. In the peri-arbuscular space, plant and fungus share a small and confined volume for the exchange of resources. In the rhizosphere, the combined efflux of organic acids and K^+, both are valuable resources for the plant, should produce a nutrient gradient that, among others, attracts beneficial microorganisms (Jacoby *et al.*, 2017).

Notes S2 The influence of ion transport on the background conductance – the K^+ battery.

In all suitable scenarios identified, the uptake of nutrients is accompanied by the transport of a positive charge. This charge transport is compensated and energised by the H^+-ATPase-driven background conductance, which in turn can be supported by the proton-coupled release of P and C via H/P and H/C symporters, respectively, which mimics the effect of proton pumping. Only if the plant replaces the H/C-transporter with SWEET, this beneficial effect is lost. The energy stored in the transmembrane sugar gradient in this case dissipates by diffusion without any further use/gain. Previously it has been shown that the flux of ions can assist the proton pump in energizing transmembrane transport processes (Dreyer *et al.*, 2017). Similar to the K^+ battery in the phloem (Gajdanowicz *et al.*, 2011; Sandmann *et al.*, 2011), the flux

of potassium ions from the fungal cytosol to the apoplast, for example, can partially replace the ATP-consuming proton pump. In our models, the description of the background conductance with its two free parameters considers these cases already. The efflux of K^+ from the fungus, for instance, is equivalent with a more negative value of the equilibrium voltage (parameter V_0) of the background conductance, while the efflux of anions corresponds to a less negative value of V_0. Thus, the screening of the background conductance parameters (Schott *et al.*, 2016) already implicitly covers all the different aspects of additional ion transport. Regarding a potassium battery, **Figs. 3c,e and Fig. S2d** just need to be interpreted in a slightly different manner: The "background ion (ATP) / P"-curves would represent the sum of hydrolysed ATP-molecules and released K^+ ions per P. This, however, refers only to the local balance. Because K^+ needs to be imported into the cytosol before at the extraradical mycelium by using the energy from ATP-hydrolysis, the K^+ release in the global balance is also equivalent to an ATP-consumption.

References in the Supporting Information

Balestrini R, Bonfante P. **2005**. The interface compartment in arbuscular mycorrhizae: A special type of plant cell wall? *Plant Biosystems* **139**: 8–15.

Dreyer I, Gomez-Porras JL, Riedelsberger J. **2017**. The potassium battery: a mobile energy source for transport processes in plant vascular tissues. *New Phytologist* **216**: 1049–1053.

Gajdanowicz P, Michard E, Sandmann M, Rocha M, Corrêa LGG, Ramírez-Aguilar SJ, Gomez-Porras JL, González W, Thibaud J-B, van Dongen JT, *et al.* 2011. Potassium (K+) gradients serve as a mobile energy source in plant vascular tissues. *Proceedings of the National Academy of Sciences of the United States of America* **108**: 864–869.

Guether M, Neuhäuser B, Balestrini R, Dynowski M, Ludewig U, Bonfante P. **2009**. A mycorrhizal-specific ammonium transporter from Lotus japonicus acquires nitrogen released by arbuscular mycorrhizal fungi. *Plant Physiology* **150**: 73–83.

Guttenberger M. **2000**. Arbuscules of vesicular-arbuscular mycorrhizal fungi inhabit an acidic compartment within plant roots. *Planta* **211**: 299–304.

Jacoby R, Peukert M, Succurro A, Koprivova A, Kopriva S. **2017**. The Role of Soil Microorganisms in Plant Mineral Nutrition—Current Knowledge and Future Directions. *Frontiers in Plant Science* **8**: 1617.

Kiers ET, Duhamel M, Beesetty Y, Mensah JA, Franken O, Verbruggen E, Fellbaum CR, Kowalchuk GA, Hart MM, Bago A, *et al.* 2011. Reciprocal rewards stabilize cooperation in the mycorrhizal symbiosis. *Science* **333**: 880–882.

Sandmann M, Skłodowski K, Gajdanowicz P, Michard E, Rocha M, Gomez-Porras JL, González W, Corrêa LGG, Ramírez-Aguilar SJ, Cuin TA, *et al.* 2011. The K^+ battery-regulating Arabidopsis K^+ channel AKT2 s under the control of multiple post-translational steps. *Plant Signaling and Behavior* **6**: 558–562.

Schott S, Valdebenito B, Bustos D, Gomez-Porras JL, Sharma T, Dreyer I. **2016**. Cooperation through competition - dynamics and microeconomics of a minimal nutrient trade system in arbuscular mycorrhizal symbiosis. *Frontiers in Plant Science* **7**: 912.

Sharma T, Dreyer I, Kochian L, Piñeros MA. **2016**. The ALMT family of organic acid transporters in plants and their involvement in detoxification and nutrient security. *Frontiers in Plant Science* **7**: 1488.
